# Reducing medication errors in neurosurgery through clinical pharmacy interventions: a prospective observational study

**DOI:** 10.1007/s10143-025-03849-8

**Published:** 2025-10-09

**Authors:** Aaron Lawson McLean, Anna Schlattl, Christian Senft, Michael Hartmann, Falko Schwarz

**Affiliations:** 1 Department of Neurosurgery, Jena University Hospital, Friedrich Schiller University Jena, Am Klinikum 1, 07747 Jena, Germany; 2 Hospital Pharmacy, Jena University Hospital, Friedrich Schiller University Jena, Jena, Germany; 3 LMU University Hospital, LMU Munich, Munich, Germany; 4 Helios Amper Hospital Dachau, Dachau, Germany

**Keywords:** Medication Errors, Neurosurgery, Clinical Pharmacy, Drug Safety, Pharmacist Interventions, Patient Safety

## Abstract

Neurosurgical patient care is inherently complex, characterized by high rates of polypharmacy, advanced age, and significant comorbidities, all of which increase the risk of medication errors. These challenges are compounded by dynamic treatment plans and intensive care demands. In response, clinical pharmacist-led “pharmaceutical interventions” have emerged as a promising strategy to enhance medication safety. This study aimed to evaluate the impact of a structured weekly pharmacist-led medication review programme on prescribing practices and patient outcomes in a tertiary academic neurosurgical department. In this 12-month prospective study, a pharmacist performed weekly medication reviews on the neurosurgical ward and HDU. Interventions were coded in ADKA-DokuPIK and relayed to the team; 10% were re-audited to confirm uptake. The year was split into two six-month epochs to assess temporal trends. Administrative data from the intervention year were compared with a historical control for length of stay (LOS) and in-hospital mortality. Adverse-drug-event rates were not prospectively collected. A total of 996 interventions were documented among 1795 patients (0.55/patient). Intervention rates declined from 0.7 to 0.4 per patient between periods (*p* = 0.016), suggesting a learning effect. Implementation of recommendations was confirmed in 78% of audited cases. The most commonly affected drugs were pantoprazole (*n* = 77), amlodipine (*n* = 47), ciprofloxacin (*n* = 44). Median LOS decreased from 8.1 to 7.3 days (*p* = 0.032), the proportion of prolonged hospitalisations (> 14 days) fell from 18.9% to 14.8% (*p* = 0.002), and in-hospital mortality declined from 4.6% to 3.0% (*p* = 0.014). Routine integration of a clinical pharmacist into neurosurgical care was associated with fewer medication-related issues, measurable improvements in LOS and mortality, and evidence of progressive prescriber adaptation. These findings support broader implementation of pharmacist-led interventions in high-risk surgical environments. Controlled multicenter trials are warranted.

## Introduction

Neurosurgical patient care is inherently complex, marked by a high burden of comorbidities [1]. Many patients are elderly and subject to polypharmacy, which in turn increases the risk of medication errors, drug–drug interactions, and dosing inaccuracies [2]. Indeed, a recent 11‐year analysis in the perioperative neurosurgical setting reported an adverse drug event rate of 18.3% across 3648 procedures, with both polypharmacy and older age emerging as independent risk factors for ADEs [3]. These challenges are further compounded by the dynamic nature of neurosurgical treatment plans and the demands of the intensive care environment [4]. In response, recent advances in patient safety have underscored the critical role of clinical pharmacists in mitigating these risks through proactive medication reviews and tailored recommendations, collectively termed “pharmaceutical interventions” [5–8].

In this study, the term “intervention” specifically refers to any pharmacist‐initiated recommendation aimed at improving the medication regimen. Such interventions include, but are not limited to, the discontinuation of drugs with unclear indications, the adjustment of dosing intervals, the correction of duplicate or erroneous prescriptions, and modifications required in response to altered renal or hepatic function. The interventions are communicated directly to the neurosurgical team during weekly integrated ward rounds, ensuring that the recommendations are considered within the clinical decision-making process. This structured approach is intended not only to prevent ADEs but also to promote a learning environment where prescribers progressively optimize their medication practices.

The present evaluation was conducted in two neurosurgical care settings at a tertiary academic neurosurgical unit. By prospectively monitoring pharmaceutical interventions over a 12‐month period, we aimed to quantify the frequency and nature of these interventions, compare rates between the normal ward and the HDU, and assess whether repeated interactions led to a measurable improvement (a “learning effect”) among prescribers. This study is grounded in the hypothesis that routine clinical pharmacist involvement will reduce the number of medication issues over time and ultimately enhance patient safety.

## Methods

### Study design and setting

We conducted a prospective observational study over a 12-month period (October 2018–September 2019) in the neurosurgical department at Jena University Hospital, the sole academic neurosurgical center in Thuringia, central Germany. The department provides continuous 24/7 coverage for both elective and emergency neurosurgical referrals across a broad supraregional catchment area. Two inpatient units were included: a 38-bed normal ward and a 10-bed high-dependency unit (HDU). This initiative formed part of a local quality improvement effort and included all patients treated on either ward, without randomization or alteration to routine clinical care. Ethical approval was granted by the institutional review board (Ethikkommission des Universitätsklinikums Jena, reference 2020–1809-Daten).

At the time of study, prescribing and documentation were managed through COPRA (COPRA System GmbH, Berlin, Germany), a clinical software system used across the department. While it records medications and known allergies, COPRA lacks real-time clinical decision support (e.g., pop-up alerts for contraindications or interactions) and does not provide automated, patient-specific prescribing guidance. As such, it does not independently flag medication-related risks during order entry, underscoring the rationale for dedicated pharmacist-led review to ensure medication safety.

### Pharmacist intervention process

A single, experienced clinical pharmacist performed a weekly ward round on each unit. Prior to each round, the pharmacist reviewed the electronic patient records using the COPRA system, with a focus on medication dosing, indications, potential interactions (using multiple drug-interaction programs), and documentation accuracy. Interventions were made when issues were identified, such as:

- Lack of a clear indication for a prescribed medication
- Potential or actual drug–drug interactions
- Contraindications based on patient-specific factors (e.g., comorbidities, concurrent therapies)
- Duplicate or erroneous prescriptions (including dosing interval errors)
- Inappropriate dosing adjustments in patients with renal or hepatic impairment

Each pharmaceutical intervention is defined as a recommendation by the pharmacist to modify the patient’s medication regimen, either by discontinuing, modifying, or substituting a drug, based on current clinical guidelines and patient-specific factors. These interventions were documented in the ADKA-DokuPIK database, a national documentation system for medication errors and interventions, using a standardized classification system [9]. Interventions were communicated directly to the treating team during weekly interdisciplinary rounds, typically involving real-time verbal discussion between the pharmacist and attending physicians or residents. The final decision on implementation of a recommendation was made by the treating neurosurgical team.

To assess implementation, a random 10% sample of the documented interventions was audited. For each sampled case, the pharmacist rechecked the patient record at the next round (within seven days). If the suggested change had been enacted, the intervention was considered “implemented”; otherwise, “not implemented.”

### Data collection and statistical analysis

Data were collected prospectively for all neurosurgical patients reviewed by the pharmacist during the 12-month intervention period. For each case, demographic information (age, sex), ward type (normal ward or HDU), and the nature and category of each pharmaceutical intervention were documented. In addition to summarizing overall intervention counts, we compared intervention rates between the two ward types to assess the impact of care setting.

To explore possible temporal effects, such as prescriber adaptation or learning, we divided the intervention period into two six-month epochs: Group 1 (October 2018 to March 2019) and Group 2 (April 2019 to September 2019). Both the normal ward and HDU operated continuously during the full study period, and the same pool of consultants, fellows, and residents rotated across both units. As a result, prescribers contributed to both care environments and both time intervals, supporting a within-group assessment of trends over time.

To explore downstream clinical impact of the interventions, we undertook a simple retrospective chart review of routine administrative data. All adult neurosurgical admissions during the 12-month baseline epoch (1 October 2017–30 September 2018) and the identical intervention epoch (1 October 2018–30 September 2019) were extracted from the hospital information system. The clinical end-points were: (1) length of stay (LOS), calculated in full days between calendar admission and discharge, and (2) in-hospital mortality, defined as death prior to discharge. These outcomes were analysed using the full census of neurosurgical admissions from both years, including short-stay cases in the intervention year not reviewed by the pharmacist because admission and discharge occurred between the pharmacist’s regular rounds.

All analyses were performed using IBM SPSS Statistics v28 (IBM Corp., Armonk, NY, USA). Continuous variables were described using medians and interquartile ranges (IQR) and compared using the Mann–Whitney U test. Categorical outcomes were analysed using Pearson’s χ^2^ test (two-tailed). No covariate adjustment was applied, in keeping with the exploratory nature of this quality-improvement initiative. A p-value < 0.05 was considered statistically significant.

## Results

### Patient population and overall intervention data

During the 12‐month study period, 1795 neurosurgical patients were evaluated by the pharmacist, and a total of 996 pharmaceutical interventions were documented (approximately 0.55 interventions per patient). The overall mean patient age was 68.4 years (SD 13.4), with patients ranging from 18 to 97 years of age. A majority of patients were in the 60–80‐year age range. A slight majority of patients were male, and the distribution of gender was similar between the normal ward and the HDU (Table 1).

**Table 1:** Patient demographics and intervention rates by ward, comparing the normal ward and the high-dependency unit (HDU)

Parameter	Normal Ward	HDU	*p*-value
Total Patients	1,370	425	—
Patients with ≥ 1 Intervention (%)	465 (33.9%)	178 (41.9%)	0.003
Median Interventions/Patient	0.5	0.7	0.022
Median Interventions/Visit	16.9	6.5	< 0.001

Administrative data identified 95 admissions, about 5% of the 1890 intervention-epoch cases, that started and finished between pharmacist rounds; the majority were elective or short-stay procedures such as lumbar decompressions or wound revisions (median LOS = 4 days), and 12 of these patients spent a single night in the HDU for observation.

### Comparison between normal ward and HDU

There were notable differences in intervention rates between the two settings. On the normal ward, 33.9% of patients required at least one intervention, whereas 41.9% of patients in the HDU received an intervention (*p* = 0.003). Moreover, the median number of interventions per patient was 0.5 on the normal ward versus 0.7 in the HDU (*p* = 0.022). In contrast, the median number of interventions per visit was significantly higher on the normal ward (16.9 per visit) compared to 6.5 per visit in the HDU (*p* < 0.001).

### Intervention frequency and distribution

Across the entire study period, 996 pharmaceutical interventions were documented, averaging approximately 0.55 interventions per patient. Table 2 shows the overall distribution of the major reasons for intervention. The most frequently encountered issue was the lack of a clear indication for medication (170 interventions, 17.0%), followed by potential drug–drug interactions (121 interventions, 12.1%), and duplicate prescriptions/dosing errors (103 interventions, 10.3%). Additional issues, such as inappropriate dosing intervals (92 interventions, 9.2%) and other medication management challenges (e.g., inadequate formulation or missing monitoring), were also noted. To assess practical uptake, a random audit of 100 interventions found that 78% had been implemented by the following week, indicating substantial integration of pharmacist recommendations into clinical practice.

**Table 2:** Frequency and distribution of key reasons for pharmacist interventions. Percentages are calculated based on the total number of interventions and represent the most common issues identified

Intervention reason	Frequency (*n*)	Percentage (%)
Lack of clear indication for medication	170	17.0
Potential drug–drug interactions	121	12.1
Duplicate prescriptions/dosing errors	103	10.3
Inappropriate dosing intervals	92	9.2

### Learning effect over time

To evaluate the impact of ongoing pharmacist involvement and any resulting “learning effect” among prescribers, the study period was divided into two equal six‐month groups. As shown in Table 3, Group 1 (October 2018-March 2019) consisted of 42 visits covering 872 patients, with a median of 14.8 interventions per visit (0.7 interventions per patient). In Group 2 (April 2019-September 2019), 44 visits with 923 patients were recorded, and the median number of interventions per visit dropped to 8.5 (0.4 interventions per patient). These differences were statistically significant (*p* < 0.001 for interventions per visit and *p* = 0.016 for interventions per patient), supporting the hypothesis that prescribers improved their medication practices over time.

**Table 3:** Comparison of intervention rates over two six-month periods to assess temporal trends: Group 1 (Oct 2018-Mar 2019) vs. Group 2 (Apr 2019-Sep 2019)

Parameter	Group 1	Group 2	p-value
Total Visits	42	44	0.930 (patients per visit)
Median Interventions/Visit	14.8	8.5	< 0.001
Median Interventions/Patient	0.7	0.4	0.016

### Most common medications affected

Table 4 summarizes the top medications for which pharmaceutical interventions were performed during the study period. The data indicate that certain medications, although routinely prescribed, were frequently subject to pharmacist review due to issues such as unclear indications, dosing errors, or potential interactions. Although some interventions involved medications of low intrinsic risk (e.g., ranitidine), many targeted agents associated with significant harm in neurosurgical populations, such as opioids, NSAIDs, anticoagulants, and QT-prolonging psychotropics.

**Table 4:** Top 10 most commonly affected medications requiring pharmacist intervention

Medication	Frequency (*n*)
Pantoprazole	77
Amlodipine	47
Ciprofloxacin	44
Metamizole	42
Ibuprofen	38
Simvastatin	36
Acetylcysteine	33
Pipamerone	31
Ranitidine	30
Etoricoxib	29

Notably, pantoprazole was the most commonly affected medication, with 77 interventions recorded. In 64 cases (83%), it had been initiated as prophylaxis during high-dose dexamethasone therapy, and in 54 of those, continued unnecessarily after steroid cessation. Given the volume of such prescriptions, routine deprescribing could yield meaningful cost reductions. Similarly, amlodipine (*n* = 47) was frequently flagged due to inappropriate use in patients with hemodynamic instability, while ciprofloxacin (*n* = 44) interventions were largely related to renal dosing adjustments or drug–drug interactions. Metamizole (*n* = 42) and ibuprofen (*n* = 38) were commonly reviewed due to concerns about nephrotoxicity or gastrointestinal risk in vulnerable patients. Additional medications with a notable frequency of interventions included simvastatin (*n* = 36), frequently due to CYP3A4-mediated interactions; acetylcysteine (*n* = 33), primarily related to unclear indications in non-respiratory conditions; and pipamperone (*n* = 31), where interventions often addressed inappropriate prescribing in elderly patients.

### Length of stay and in-hospital mortality

Retrospective review of routine administrative data demonstrated that the pharmacist-led programme was associated with modest but statistically significant improvements in patient-centered outcomes (Table 5). Median LOS fell from 8.1 days (IQR 6–13) in the 2017–18 baseline cohort to 7.3 days (IQR 5–12) in 2018–19 (*p* = 0.032). The proportion of admissions exceeding 14 days decreased from 18.9% to 14.8% (*p* = 0.002). Crude in-hospital mortality likewise declined, from 78/1689 admissions (4.6%) to 57/1890 admissions (3.0%; *p* = 0.014). An additional 95 short-stay admissions during the intervention epoch were not pharmacist-reviewed; sensitivity analysis including these cases did not materially alter LOS or mortality estimates.

**Table 5:** Patient-centred outcomes (LOS and in-hospital mortality) before and after pharmacist intervention

Outcome	Baseline 2017–18 (*n* = 1689)	Intervention 2018–19 (*n* = 1890)	*p*-value
Median LOS, days (IQR)	8.1 (6–13)	7.3 (5–12)	0.032
Prolonged LOS > 14 d, *n* (%)	320 (18.9%)	280 (14.8%)	0.002
In-hospital mortality, *n* (%)	78 (4.6%)	57 (3.0%)	0.014

## Discussion

Our 12-month evaluation suggests that embedding a dedicated clinical pharmacist within the neurosurgical team not only enables the early identification and correction of medication-related problems but also fosters a learning effect among prescribers. These changes coincided with measurable improvements in patient outcomes, including a reduction in median LOS and crude in-hospital mortality.

Specifically, median LOS decreased by 0.8 days (from 8.1 to 7.3), and the proportion of prolonged hospitalisations (> 14 days) declined significantly. In-hospital mortality fell from 4.6% to 3.0%, representing a relative reduction of approximately 35%. While these findings are exploratory and unadjusted, they suggest that safer and more appropriate prescribing may translate into clinically relevant benefit. Notably, these associations persisted even when 95 short-stay cases not directly reviewed by the pharmacist were included in a sensitivity analysis, arguing against a sampling artefact and suggesting a broader system-level effect.

The higher intervention rate observed in the HDU relative to the normal ward is consistent with the greater clinical complexity and medication burden seen in critically ill neurosurgical patients [10, 11].

The significant decline in intervention rates from Group 1 to Group 2 may reflect a learning effect, potentially indicating improved prescribing practices and closer collaboration between the pharmacist and neurosurgical staff. It is plausible that prescribers began to incorporate the pharmacist’s recommendations into their routine decision-making, resulting in fewer identified issues over time. While causality cannot be confirmed, this pattern is consistent with the hypothesis that sustained interprofessional engagement can enhance medication management and reduce the risk of ADEs. Similar trends have been reported in other clinical contexts, where iterative feedback and regular pharmacist input have been associated with improvements in prescribing quality and reductions in drug-related problems [12–15]. The structured process of reviewing medication regimens and offering targeted feedback may contribute to more informed prescribing and, by extension, improved patient safety.

Pantoprazole was the most frequently intervened-upon medication (*n* = 77), suggesting potential overuse or suboptimal indication of proton pump inhibitors in neurosurgical patients. Other frequently flagged agents included amlodipine, ciprofloxacin, metamizole, and ibuprofen. Our findings align with observations by Weber et al., who similarly identified pantoprazole as a common non‐anticancer drug implicated in medication errors in the oncology setting, highlighting that even low-risk, routinely prescribed medications are susceptible to inappropriate continuation, misdosing, or harmful interactions. [16].

In neurosurgery, such risks are amplified by the frequent use of agents with narrow therapeutic indices or steep dose–response curves, such as antiepileptics, hyperosmolar agents, and anticoagulant reversal drugs. These medications often require careful titration, renal or weight-based dosing, and close monitoring. Abrupt initiation or withdrawal, common around surgery, can precipitate adverse effects or therapeutic failure. High-dose corticosteroids further complicate regimens through metabolic and immunosuppressive side effects, and are often prescribed without clear tapering plans. The dynamic neurological status of patients, coupled with time-sensitive decision-making and rotating prescriber teams, may reduce opportunities for comprehensive medication reconciliation. These features create a uniquely error-prone prescribing environment, where structured pharmacist review can support timely identification of contraindications, duplications, or inappropriate continuation and help mitigate avoidable harm.

In the neurosurgical setting, dedicated pharmacist-led interventions remain underexplored compared with other surgical specialties. Notably, Greenwood et al. conducted a pilot study demonstrating that a pharmacy discharge review in neurosurgical patients can improve medication safety and care quality [17]. Similar benefits have been observed in related fields: for instance, pharmacist-led interventions in vascular surgery have significantly reduced drug-related problems, and in cancer surgery, comprehensive medication management from preadmission to discharge has been associated with improved outcomes [15, 18]. Moreover, interdisciplinary models implemented in primary care and elderly care, such as the IMMENSE study and targeted deprescribing initiatives, have consistently demonstrated reductions in medication errors and enhanced patient adherence [14, 19]. Collectively, these related studies underscore the potential value of extending structured, pharmacist-led medication reviews into neurosurgical care to address the high complexity and polypharmacy prevalent in this population.

A full formal cost-effectiveness analysis was beyond the scope of this observational study and is acknowledged as a limitation. Nevertheless, local salary data allow an illustrative budget impact. The pharmacist’s fully loaded employer cost (i.e., salary plus all mandatory hospital contributions for health, pension and unemployment insurance, as well as payroll taxes) was €5612.77 per month. At 5 h weekly for the normal ward and 3 h for the HDU (8 h total, 20.4% of a full-time post), the annual labour cost of the programme was therefore €5 612.77 × 12 × 0.204 ≈ €13 700. Spread over the 996 documented interventions this equals €13.7 per intervention or €17.6 per implemented recommendation once the 78% uptake rate is applied. No extra software or consumable costs were incurred.

To gauge return on investment it is possible to draw on published German and French costing studies. A micro-costing analysis of 564 ADE-related admissions in German hospitals reported a mean direct cost of €1978 (median €1446; range €191–18147) per case [20]. A more recent French evaluation placed the mean cost of an ADR-related admission at €5974 under 2023 tariffs [21]. On this evidence, a conservative working range of €3000–5000 per preventable event is reasonable. Avoiding just three such events per year would offset the programme’s €13700 annual cost; any additional prevention would generate net savings.

These findings are in line with prior evaluations suggesting that integrating clinical pharmacists into intensive care settings can yield substantial cost benefits. For instance, an economic evaluation in Potsdam, Germany, demonstrated that 177 pharmacist-led interventions over an 8‑month period prevented approximately 16 ADEs, translating to an estimated annual saving of nearly €80,000 (approximately 85,000 USD at 2024 exchange rates) [22]. This figure may underestimate the true economic advantage, as additional savings from optimized therapy and reduced complications were not captured. Comparable financial benefits have been reported in other studies. For example, Nesbit et al. and Wei et al. demonstrated significant cost savings from a clinical pharmacist practice model [23, 24]. Likewise, Dawoud et al. confirmed the cost-effectiveness of pharmacist input at the ward level through a systematic review and meta-analysis. Further, Leape et al. showed that pharmacist participation on physician rounds notably reduced ADEs, with implications for lowered healthcare expenditures [25, 26].

### Strengths and limitations

This study contributes to the growing evidence base from critical care and oncology by extending pharmacist-led interventions into neurosurgery, a specialty where such participation remains uncommon. It demonstrates that even a straightforward model with remote chart review followed by a structured weekly ward round can reliably identify prescribing problems and may be associated with improved outcomes, including shorter hospital stays and lower in-hospital mortality.

These findings should be interpreted with caution. The study was conducted in a single academic hospital with one clinical pharmacist; institutions with different cultures, electronic prescribing systems, or staffing models may not observe similar outcomes. The absence of a contemporaneous control ward limits causal inference; observed reductions in intervention frequency, length of stay, and mortality cannot be confidently attributed to a pharmacist-driven learning effect, as unmeasured factors (such as staff turnover, case-mix variation, or concurrent quality improvement efforts) may have contributed. The weekly review schedule means that prescribing errors arising between rounds could persist for several days, and 95 short-stay admissions were never directly reviewed by the pharmacist, meaning outcome denominators included patients who did not receive the intervention. We did not capture individual prescriber seniority or prior experience, so residual confounding of temporal trends is possible. Lastly, recommendation uptake was assessed retrospectively from a 10% sample and showed that approximately three-quarters of suggestions were implemented by the next round; prospective, real-time assessment would provide a more accurate measure. Multicenter controlled studies with risk adjustment and validated clinical endpoints are needed to determine whether the associations observed here reflect a causal and generalizable benefit of pharmacist integration.

### Generalizability and scalability

The intervention in this study, one pharmacist who reviews charts remotely and attends a scheduled weekly multidisciplinary ward round, relies on three elements that are readily exportable: (i) basic electronic access to each patient’s medication list, (ii) a ward culture that welcomes pharmacist input during rounds, and (iii) a predictable, modest allocation of pharmacist time. In hospitals where full on-site cover is impractical, the same framework could be adapted through options such as brief scheduled visits or tele-pharmacy participation. These institutional factors, rather than academic status or sophisticated IT infrastructure, are likely to determine whether similar benefits can be realised in non-academic or resource-constrained settings.

## Conclusions

In this study, the implementation of a structured weekly pharmacist-led medication review in a neurosurgical department was associated with a significant reduction in medication-related interventions over time. The data suggest that pharmacist involvement not only directly improves medication safety through targeted interventions but also contributes to a learning effect among neurosurgical prescribers. These findings support the further integration of clinical pharmacy services into neurosurgical care, with potential implications for improved patient outcomes and cost savings. Future studies should evaluate the long-term clinical impact and explore the synergistic role of artificial intelligence-based decision support systems to complement human oversight and provide continuous medication safety monitoring.

## Data Availability

The datasets generated and analyzed during the current study are not publicly available due to institutional data protection policies and patient confidentiality constraints, but are available from the corresponding author on reasonable request and with appropriate ethical approval.
